# Dr. Gillespie, on the Bites of Serpents

**Published:** 1800-10

**Authors:** Leonard Gillespie

**Affiliations:** Naval Hospital, Fort Royal, Martinique


### *To the Editors of the Medical and Phyſical Journal.*

GENTLEMEN,

FATAL accidents from the bites of venomous ſerpents are far from being uncommon in the iſland of Martinique; two inſtances of the ſort have happened in the neighbourhood of this city within the laſt ſix months, terminating in death in twenty-four hours after the accident.

It would ſeem inexplicable, from natural cauſes, why Martinique, St. Lucia, and (according to ſome) the ſmall iſland of Bequice ſhould, of all the numerous iſlands compoſing this Archipelago, be infected with theſe venomous reptiles. Le Pere du Tertre, in his excellent Hiſtory of the Antilles, Vol. ii. p. 319, relates a tradition of the native Caribbes, as to the origin of venomous ſerpents in Martinique and St. Lucia: They informed this Hiſtorian, that their forefathers waging a war of extermination with the Arrouagues, a nation of Terra Firma, the latter, unequal in war to their Caribb enemies, who made frequent incurſions upon their coaſts, bethought themſelves of a ſingular expedient to be revenged on the iſlanders. They collected in baſkets and earthen pots a number of venomous ſerpents, which, tranſporting to Martinique, there gave them their liberty, in order that, without departing from their own coaſt in future, they, the Arrouagues, might make an eternal war againſt their Caribb enemies.'

However this may be, Nature has not abandoned man without remedy againſt the deadly venom of this reptile, the fecundity of which is extraordinary, it not being uncommon to find ſixty or ſeventy young ones in the belly of a female, of which I have been an ocular witneſs in one caſe. The negroes long accuſtomed to the iſland, have not, in general, much apprehenſion from the bite of a ſerpent, (I have known ſeveral who have been bit repeatedly) whilſt thoſe who come here from the other iſlands, have as much dread of theſe reptiles as the Europeans. Two inſtances of perſons bit by venomous ſerpents have occurred here, both of whom recovered. The firſt was John Payne, a negro, reported to be in a dying ſtate about noon of Feb. 20, 1796. On viſiting him, he was found to be affected with the moſt dreadful convulſions, each acceſſion of which threatened to terminate his exiſtence. He had been bitten the night before, but being exceedingly intoxicated he had not paid any attention to it. When the effect of the ſpirits he had taken went off, he found his arm ſtiff, painful, and ſwelled up to the axilla; to theſe ſymptoms quickly ſucceeded nauſea, laborious reſpiration, and convulſions. The marks of the venomous fangs were obſervable over the abductor policis of the left hand, which, with the arm and breaſt, was conſiderably inflated. The patient was inſenſible for ſome time after every convulſion; and when ſenſible, he expreſſed the utmoſt dread of inſtant diſſolution. His anxiety was great, his pulſe was ſlow, ſmall, and irregular. Topical treatment of the envenomed points was judged uſeleſs after a lapſe of fourteen hours, the lymphatic glands and veſſels being evidently ſtrongly affected; the limb and breaſt were, however, bathed with hot lime juice and rubbed with camphorated Caſtor oil. A large doſe of Venice treacle with volatile aromatic ſpirit and vitriolic æther was adminiſtered to the patient, waſhed down with warm rum and water, which was repeatedly given him in ſmall quantities. After ſeveral frightful convulſion fits, his pulſe began to riſe, the reſpiration became more free, ſigns of a diaphoreſis appeared, and hope began to influence his mind. A ſecond doſe of the medicine was given him with the hot drink, and he was well covered with hot blankets; about two o'clock he turned himſelf on his ſide, fell into a warm ſweat, got ſome refreſhing ſleep, and was perfectly well next day.

The ſecond Caſe was that of Utill, a negro, who has often experienced the like accident. March 26th ult. he was bitten by a venomous ſerpent in the middle and external part of the left fore arm, whilſt cutting graſs near to the country-houſe of Admiral Lord Hugh Seymour, ſituated a mile from this town. His Lordſhip being near the ſpot, immediately made a ligature on the limb, applied fome fpirit of hartfhorn to the wounds, gave the negro fome of the fame internally, mixed with rum and water, and fent him off to this Hofpital. The punctures made by the poifonous fangs were evident; the arm was painful, and a little fwelled up to the axilla; and the patient, tho' undifmayed, (a matter of great confequence in fuch circumftances) experienced fome anxiety and pain of the left breaft. Several fcarifications were inftantly made around the punctures, over which a cupping-glafs was applied, and three or four ounces of blood extracted; the part was then fpunged with warm water, the limb and breaft were bathed with Caftor oil, and a fmall poultice was applied to the part, compofed of roafted limes, a little falt and black pepper, and fome of the powdered leaves of a fpecies of Ariftolochia (*Anguicida, Lin.*) the Lianne, a ferpent of the French iflands. Some [illegible] with fpirit of hartfhorn was given him internally, and he took fome of the infufion of the Ariftolochia internally. He was prevented from going to fleep, to which he feemed difpofed, and which is faid to be very dangerous in fuch circumftances, and remained exempt from any accident.

It is here proper to obferve, that the Ariftolochia was ufed externally and internally in this Cafe, on account of the ftrong impreffion on the mind of the negro, of the fpecific power of that fimple againft the venom of the ferpent, rather than from any rational dependance on it; as however effectual it may be when ufed frefh and fucculent, that applied in this inftance feemed nearly infipid from having been long dried and carried in the pocket of the patient as an amulet, according to the fuperftition of the negroes. Befides the fnake-wood, or Ariftolochia, in moft general ufe by the negroes here againft venomous bites, there is a fpecies of grafs, called Pie de Poule, or chicken-foot, which grows very commonly here; this, beat up with a little falt and fpirits, is applied as a remedy to venomous bites. Of this I have had no experience; but I have often ufed this fimple with much benefit as a detergent and antifeptic in foul ulcers. Arfenic and the volatile alkali are faid to be ufed by fome with falutary effects againft thefe accidents; wood afhes and oil are alfo faid to be ufed in the fame intention: This is recommended by the Pere du Tertre, as well as a poultice of quick-lime with oil and honey; garlic externally applied, and given internally; the green leaves of tobacco, bruifed, and applied to the part; whilft theriaca and cordials are to be adminiftered; and the patient is to be reftrained from taking large draughts of any liquid, or from the dangerous indulgence of fleeping, to which there is generally much propenfity. I am, GENTLEMEN,

Your moft obedient fervant,

LEONARD GILLESPIE, M. D.

*Naval Hospital, Fort Royal, Martinique,*
*May 7,* 1800.

